# Transient Receptor Potential Ankyrin-1-expressing vagus nerve fibers mediate IL-1β induced hypothermia and reflex anti-inflammatory responses

**DOI:** 10.1186/s10020-022-00590-6

**Published:** 2023-01-18

**Authors:** Harold A. Silverman, Aisling Tynan, Tyler D. Hepler, Eric H. Chang, Manojkumar Gunasekaran, Jian Hua Li, Tomás S. Huerta, Tea Tsaava, Qing Chang, Meghan E. Addorisio, Adrian C. Chen, Dane A. Thompson, Valentin A. Pavlov, Michael Brines, Kevin J. Tracey, Sangeeta S. Chavan

**Affiliations:** 1 Laboratory for Biomedical Sciences, Institute for Bioelectronic Medicine, Feinstein Institutes for Medical Research, Northwell Health, 350 Community Drive, Manhasset, NY 11030 USA; 2 Donald and Barbara Zucker School of Medicine at Hofstra/Northwell, 500 Hofstra Blvd, Hempstead, NY 11549 USA; 3 Department of Surgery, North Shore University Hospital, Northwell Health, 300 Community Drive, Manhasset, NY 11030 USA; 4 The Elmezzi Graduate School of Molecular Medicine, Northwell Health, 350 Community Drive, Manhasset, NY 11030 USA

**Keywords:** Neural anti-inflammatory response, TRPA1, Cytokines, Nociception

## Abstract

**Background:**

Inflammation, the physiological response to infection and injury, is coordinated by the immune and nervous systems. Interleukin-1β (IL-1β) and other cytokines produced during inflammatory responses activate sensory neurons (nociceptors) to mediate the onset of pain, sickness behavior, and metabolic responses. Although nociceptors expressing Transient Receptor Potential Ankyrin-1 (TRPA1) can initiate inflammation, comparatively little is known about the role of TRPA1 nociceptors in the physiological responses to specific cytokines.

**Methods:**

To monitor body temperature in conscious and unrestrained mice, telemetry probes were implanted into peritoneal cavity of mice. Using transgenic and tissue specific knockouts and chemogenetic techniques, we recorded temperature responses to the potent pro-inflammatory cytokine IL-1β. Using calcium imaging, whole cell patch clamping and whole nerve recordings, we investigated the role of TRPA1 during IL-1β-mediated neuronal activation. Mouse models of acute endotoxemia and sepsis were used to elucidate how specific activation, with optogenetics and chemogenetics, or ablation of TRPA1 neurons can affect the outcomes of inflammatory insults. All statistical tests were performed with GraphPad Prism 9 software and for all analyses, P ≤ 0.05 was considered statistically significant.

**Results:**

Here, we describe a previously unrecognized mechanism by which IL-1β activates afferent vagus nerve fibers to trigger hypothermia, a response which is abolished by selective silencing of neuronal TRPA1. Afferent vagus nerve TRPA1 signaling also inhibits endotoxin-stimulated cytokine storm and significantly reduces the lethality of bacterial sepsis.

**Conclusion:**

Thus, IL-1β activates TRPA1 vagus nerve signaling in the afferent arm of a reflex anti-inflammatory response which inhibits cytokine release, induces hypothermia, and reduces the mortality of infection. This discovery establishes that TRPA1, an ion channel known previously as a pro-inflammatory detector of cold, pain, itch, and a wide variety of noxious molecules, also plays a specific anti-inflammatory role via activating reflex anti-inflammatory activity.

**Supplementary Information:**

The online version contains supplementary material available at 10.1186/s10020-022-00590-6.

## Background

A species survives when its individual members overcome dual threats from pathogen invasion (infection) and tissue damage (injury). This evolutionary pressure produced host defense systems which detect molecular products of microbes and injury and initiate physiological responses to repel pathogens and activate tissue repair. But these same inflammatory responses carry risk because unrestrained inflammation is self-amplifying and capable of collateral damage to normal tissues. This occurs when cytokines, proteins released by immune-competent cells to coordinate inflammation, are produced in large amounts necessary and sufficient to mediate shock, tissue injury, and lethal organ damage (Kany et al. 2019). Accordingly, evolution has conferred vertebrates with homeostatic anti-inflammatory control mechanisms that inhibit inflammation by regulating cytokine production (Andersson and Tracey 2012; Brines and Cerami 2008).

The onset of infection and injury stimulates sensory neurons to relay action potentials in the central nervous system (CNS). In the brainstem the arrival of these incoming signals initiates reflex outputs from the brain which return to the peripheral organs via the autonomic nervous system and the hypothalamus–pituitary–adrenal axis. These neural and endocrine signals attenuate pro-inflammatory cytokine production to prevent cytokine storm and maintain homeostasis. A well-defined neural mechanism is mediated by the vagus nerve which occupies a central role in mediating these protective anti-inflammatory responses (Tracey 2002) via signals descending from the brainstem which inhibit cytokine production, attenuate inflammation, and prevent the onset of tissue damage in animal models of shock, arthritis, ischemia, colitis, and other conditions. Recent and ongoing clinical studies show that vagus nerve stimulating devices which activate a reflex anti-inflammatory response in humans also inhibit cytokine production and tissue damage in patients with rheumatoid arthritis, inflammatory bowel disease, and other conditions (Aranow et al. 2021; Koopman et al. 2016; Tynan et al. 2022; Bonaz et al. 2017). The neural anti-inflammatory mechanisms which inhibit cytokines have been revealed at the level of brainstem nuclei, vagus nerve anatomy, and cholinergic signal transduction in cytokine producing cells, but it had remained mysterious how infection and injury initiates this response.

Peripheral sensory neurons, termed nociceptors, express several families of voltage-gated ion channels which initiate and propagate sensory input as action potentials. The Transient Receptor Potential (TRP) channel family, including TRP Ankyrin 1 (TRPA1), has been implicated in both activating and inhibiting inflammatory responses (Bautista et al. 2013). TRPA1 is an evolutionarily ancient channel, widely expressed by somatosensory neurons in species as varied as worms and mammals. TRPA1 nociceptors are activated by diverse chemicals, reactive oxygen species, electrophilic compounds, temperature changes, and inflammatory mediators (Laursen et al. 2014), and also by lipopolysaccharide (LPS), a bacterial endotoxin produced during infection. LPS-induced TRPA1 channel activation in nociceptive sensory neurons mediates neurogenic inflammation independently of TLR4 activation, the endogenous LPS receptor (Meseguer et al. 2014). LPS also activates monocytes and macrophages to produce interleukin-1 beta (IL-1β), an early cytokine mediator in murine injury and infection which mediates hypothermia, cytokine storm, and inflammation (Mantovani et al. 2019; Dinarello et al. 2012). Recently, Soni et al. observed that IL-1 stimulates calcium uptake in glomerular mesangial cells by a mechanism that requires TRPA1 (Soni et al. 2021), and we independently observed that IL-1β applied to the vagus nerve stimulated afferent compound action potential waveforms (Steinberg et al. 2016; Zanos et al. 2018).

As recent work has shown that direct activation of TRPA1 channels of the vagus and trigeminal nerves elicits hypothermia (Matsuo et al. 2021a), here we reasoned that IL-1β activation of TRPA1-expressing vagus nociceptors mediates hypothermia and provides sensory input to initiate vagus nerve mediated reflex anti-inflammatory responses. Administration of IL-1β to mice activated TRPA1-expressing nociceptors in the vagus nerve, induced the onset of hypothermia, and stimulated an anti-inflammatory response, a homeostatic mechanism which inhibited cytokine storm during endotoxemia, and significantly improved survival from lethal sepsis.

## Methods

### Animals

All procedures with experimental animals were approved by the Institutional Animal Care and Use Committee and the Institutional Biosafety Committee of the Feinstein Institutes for Medical Research, Northwell Health, Manhasset, NY in accordance with the National Institutes of Health Guidelines. C57 BL/6, B6.129PF, TRPV1 knockout (KO) (B6.129X1-*Trpv1*^*tm1Jul*^/J), TRPA1 KO (B6;129P-*Trpa1*^*tm1Kykw*^/J), α7nAChR KO (B6.129S7-Chrna7^tm1Bay/J^), Syn-Cre (B6.Cg-Tg(Syn1-Cre) 671Jxm/J), TRPA1^fl/fl^ (129S-*Trpa1*^*tm2Kykw*^/J), Gq-DREADD (B6N;129-Tg(CAG-CHRM3*,-mCitrine)1Ute/J), Vglut2-ires-Cre (*Slc17a6*^*tm2(cre)Lowl*^/J), and Rosa26-GCaMP3 (B6.Cg-*Gt (ROSA)26Sor*^*tm38(CAG-GCaMP3)Hze*^/J), mice were purchased from Jackson Laboratory (Jackson Laboratory, Bar Harbor, ME, USA). TRPA1-Cre-DTR mice were a generous donation from Columbia University (C. Zuker laboratory). Syn-Cre female mice were bred with TRPA1^fl/fl^ male mice to generate heterozygous F1 generation Syn-Cre/TRPA1^fl/+^ mice. F1 generation Syn-Cre/TRPA1^fl/+^ mice were then back crossed to TRPA1^fl/fl^ mice to create an F2 generation of Syn-Cre/TRPA1^fl/fl^ mice to be used for experiments (Additional file 1: Fig. S2). TRPA1-Cre-DTR mice were bred with IL1R1^fl/fl^ mice to generate TRPA1-Cre/IL1R1^fl/+^ mice. F1 generation TRPA1-Cre/IL1R1^fl/+^ mice were then back crossed to IL1R1^fl/fl^ mice to create an F2 generation of TRPA1-Cre/ IL1R1^fl/fl^ mice to be used for experiments (Additional file 1: Fig. S2). TRPA1-Cre-DTR mice were bred with Gq-DREADD mice to generate TRPA1-Cre/Gq-DREADD mice. VGlut2-ires-Cre mice were bred with Rosa26-GCaMP3 mice to generate Vglut2-Cre/GCaMP3 mice that express GCaMP3 allele under control of the *Vglut2* locus. The genotypes of the transgenic strains were confirmed using PCR (Transnetyx, Cordova, TN). For these experiments male mice were used as these exhibit a more reliable and severe inflammatory response to endotoxin (Cai et al. 2016). For vagus nerve recording experiments, food was withheld for the 3–4 h prior to recording; animals continued to have access to water.

### Telemetry system for temperature recordings

Mice were anesthetized using isoflurane at 2.5% in 100% oxygen at a flow rate of 1 L/min and maintained in supine position at 2.0% isoflurane. A midline incision was made, and an ETA-F10 temperature implant (DSI New Brighton, MN) was placed in the peritoneal cavity tacked to the peritoneal wall. After a minimum 5-day recovery period, mice were placed onto the DSI receiver. Baseline body temperature and relative activity was recorded for 1 h prior to intraperitoneal injection of IL-1β (0.5 µg/kg, 5 µg/kg, 50 µg/kg), saline (8.0 µL/g), clozapine-N-oxide (5 mg/kg), vehicle, or TNF (0.4 mg/kg). Recordings were continued for up to 7 h post injection during their active cycle (lights off). Animals were housed and experiments conducted in a precision temperature-controlled environment assuring constant ambient temperature. A baseline was established at the time of injection (time zero) for area under the curve (AUC) analysis.

### Electrophysiological recording

The vagus nerve recordings were performed as described previously (Zanos et al. 2018; Silverman et al. 2018). Briefly, mice were anesthetized using isoflurane at 2.5% in 100% oxygen at a flow rate of 1 L/min, and maintained in supine position at 1.5% isoflurane on a heating pad to maintain core body temperature around 37 °C. The cervical vagus nerve was then exposed and placed on the recording electrode. Recordings were sampled at 40 kHz with a 120 Hz filter and 1:50 gain at 1.25% isoflurane. Electrophysiological signals were recorded using a bipolar cuff electrode (CorTec, Freiburg, Germany) referenced to the animal ground placed between the right salivary gland and the skin. Following acquisition of the baseline activity (5 min), 350 ng/kg recombinant human IL-1β was administered intraperitoneally; recordings were then continued for 5 min post-injection. The electrophysiological signals were digitized from the vagus nerve using a Plexon data acquisition system (OmniPlex, Plexon Inc., Dallas, Texas) and analysed using Spike2 software (version 7, CED) as described previously (Zanos et al. 2018).

### Opto-pharmacological stimulation of TRPA1 positive fibers on the vagus nerve

Mice were anesthetized and the left cervical vagus nerve was surgically exposed as described above. Optovin (2 µL of 15 mM solution) was directly applied on the nerve 2 min prior to light stimulation. Animals were subjected to light stimulation (1000 mA, 10 Hz, with a 10% duty cycle for 5 min) using Thor Labs LED driver DC4100, with a 405 nm LED model M405L3 (Newton, New Jersey). Sham stimulation group underwent similar surgical procedure and optovin application, but no light stimulation. Following light stimulation, the wound was approximated in two layers using 5-0 vicryl sutures (Ethicon, Somerville, NJ, USA) and skin clips. Mice were allowed to recover on a heat pad prior to endotoxemia.

### Endotoxemia

LPS (*Escherichia coli* 0111:B4; Sigma; 1 mg/mL in saline) was sonicated for minimum of 30 min and administered intraperitoneally as indicated. Mice subjected to light or sham stimulation were allowed to recover for 2 h prior to LPS administration. In experiments with TRPA1 agonist administration, mice received polygodial (Tocris, Minneapolis, MN, USA) (5 mg/kg, i.p.) or vehicle (i.p.) 30 min prior to LPS administration (0.1 mg/kg). TRPA1-Cre/Gq-DREADD mice were subjected to either chemogenetic stimulation receiving either clozapine N-oxide (Tocris, Minneapolis, MN, USA) at 5 mg/kg (i.p.) or vehicle (i.p.) 1 h prior to LPS administration (0.3 mg/kg i.p.); or direct application of clozapine N-oxide (CNO, 0.25 mg/kg) or vehicle to the vagus nerve for 5 min. Following direct application of clozapine N-oxide to the vagus nerve, mice were recovered for 2 h prior to LPS (1.0 mg/kg). Animals were euthanized by CO_2_ asphyxiation 90 min post-LPS administration and blood was collected by cardiac puncture. Serum TNF levels were quantitated using commercial enzyme-linked immunosorbent assay (ELISA) (Invitrogen, Thermofisher Scientific, Waltham, MA, USA).

### Vagotomy

A 6.0 suture was tied around the vagus nerve, and a surgical cut of the left cervical vagus nerve was completed either rostral or caudal to the optovin application site, using the brain as the point of reference. Animals were then subjected to optovin application, light stimulation and endotoxin administration as previously described.

### Neuronal cultures

Nodose ganglia from B6.129PF, TRPA1 KO or Vglut2-Cre/GCaMP3 were dissected into ice-cold neurobasal medium (Gibco, Thermo Fisher Scientific), and dissociated with 1 µg/mL collagenase/dispase (Roche Life Science, Germany) for 90 min at 37 °C on a rotator-shaker. After trituration using to dissociate intact nodose ganglia, cells were filtered with a 70 µm nylon cell strainer, and centrifuged. Cells were plated on poly-l lysine (100 µg/mL, Sigma-Aldrich, St. Louis, MO) and laminin (50 µg/mL, Sigma-Aldrich) coated glass cover slips in 24 well tissue culture plate in complete neurobasal medium [Neurobasal™ medium supplemented with penicillin–streptomycin (Gibco), GlutaMax™ (Gibco), B-27® serum-free supplement (Gibco), 50 ng/mL NGF (Sigma-Aldrich)], and allowed to adhere for 24–48 h at 37 °C (with 5% CO_2_) prior to proximity ligation assay and intracellular calcium measurements.

### Calcium imaging

Sensory nodose ganglion neurons isolated from wildtype (B6.129PF) and TRPA1 KO mice were loaded with Fluo-4 NW with pluronic acid F-127 (Molecular Probes) for 60 min at 37 °C for 45 min in neurobasal medium, washed and imaged at room temperature. For imaging the nodose ganglion neurons from VGlut2-Cre/GCaMP3 mice, cells were isolated, cultured on coverslips as described above and imaged. Confocal images were acquired continuously at a frame rate of 4 Hz with an imaging resolution of 512 × 512 pixels while chemical agonists 20 µg/mL IL-1β, 100 µM AITC (Sigma-Aldrich), 10 µM polygodial, and 10 µM capsaicin (Sigma-Aldrich) were applied via a valve-controlled perfusion system (Warner Instruments) or pipette on a Zeiss LSM-880 confocal laser microscope. 1-[[3-[2-(4-chlorophenyl)ethyl]-1,2,4-oxadiazol-5-yl]methyl]-1,7-dihydro-7-methyl-6*H*-purin-6-one (AM0902; Tocris, Minneapolis, MN, USA) was perfused as indicated in respective experiments. Cells were washed with HBSS before application of each new agonist. Individual neurons were identified and analyzed for fluorescence intensity changes offline.

### Ex vivo intact nodose ganglion imaging

For ex vivo imaging, intact nodose ganglia along with a part of the cervical vagus nerve were excised from VGlut2-Cre/GCaMP3 mice, placed into HBSS buffer, and mounted into a custom-fabricated glass holding chamber. This chamber is designed to sit within a standard liquid perfusion system (Warner Instruments, Hamden, CT, USA) mounted onto the imaging stage of a Zeiss LSM-880 confocal laser microscope. Confocal images were acquired continuously at a frame rate of 4 Hz with an imaging resolution of 512 × 512 pixels while chemical agonists were applied via a valve-controlled perfusion system (Warner Instruments). Neurons were challenged with IL-1β (20 µg/mL), followed by polygodial (200 µM) and capsaicin (10 µM). Cells were washed with HBSS before application of each new agonist. Individual neurons were identified and analyzed for fluorescence intensity changes offline using ImageJ.

### Whole-cell patch-clamp recordings

Whole-cell patch-clamp recordings were performed as described previously (Chang and Martin 2016). Nodose ganglia neurons from Vglut2-Cre/GCaMP3 mice were selected for recording by their calcium fluorescence response to batch application of IL-1β (20 µg/mL). For patch-clamp recordings, neurons were visualized on a SliceScope system (Scientifica, Thermo Fisher Scientific) with an Olympus BX51 microscope and Luigs and Neumann micromanipulators. Glass electrodes had a resistance of 2–4 MΩ and contained an intracellular solution (in mM): KCl 140, HEPES 10, EGTA 5, Mg-ATP 2, NaGTP 0.3, MgCl_2_ 2, phosphocreatine 10, pH 7.25 adjusted with KOH. The cells were perfused at a rate of 2 mL/min at room temperature (20–22 °C) with an external bath solution containing the following (in mM): 140 NaCl, 5 KCl, 10 HEPES, 10 d-glucose, 2 CaCl_2_, and 1 MgCl_2_, pH 7.3–7.4. Whole-cell currents were acquired using an Multiclamp 700B amplifier (Molecular Devices, Union City, CA) and Windows PC running pClamp 11 software (Molecular Devices). Recordings were filtered at 2 Hz and sampled at 10 Hz. Data was analyzed offline using Clampfit 11 (Molecular Devices) and Origin 2019.

### Duolink proximity ligation assay (PLA)

The interactions between TRPA1 and IL1R1 in nodose ganglia cultures were detected by PLA with Duolink in situ kit (Sigma-Aldrich). Primary nodose ganglion cells were harvested and cultured on glass coverslips as described above. The cells were fixed with 4% paraformaldehyde/PBS for 15 min, washed with PBS, permeabilized with 0.2% Triton X-100 for 15 min, blocked with Duolink blocking solution for 60 min at 37 °C, and then incubated in primary antibodies diluted in Duolink antibody diluent overnight at 4 °C. Primary antibodies were rabbit anti-TRPA1 (polyclonal, 1:500; Millipore, Temecula, CA) and goat anti-IL1R1 (polyclonal, 1:40; R&D Systems). After incubation with primary antibodies, cells were washed and then incubated for 60 min at 37 °C in Duolink anti-goat PLUS and anti-rabbit MINUS PLA probe solutions. The coverslips were then washed and incubated with the ligation solution for 30 min at 37 °C. After ligation, the cells were washed and incubated with amplification solution for 100 min at 37 °C. After washing, cells were mounted using Duolink mounting medium with DAPI. Labeled cells were visualized and imaged using a confocal microscope (Zeiss LSM 880). The resulting positive signals were recognized as discrete fluorescent spots. Each spot represents one interaction event. All specimens were imaged under identical conditions and analyzed using identical parameters.

### Cecal ligation and puncture

A standardized model of cecal ligation and puncture (CLP)-induced sublethal polymicrobial sepsis was used as previously described (Yang et al. 2015). Mice were anesthetized using ketamine (100 mg/kg, i.p.) and xylazine (8 mg/kg, i.p.). The cecum was isolated and ligated below the ileocecal valve and then punctured with a 22-gauge needle. Approximately 2 mm of stool was then extruded, with the cecum returned to the abdominal cavity. The abdomen was closed with surgical clips. An antibiotic, Primaxin (Imipenem–Cilastatin, 0.5 mg/kg, subcutaneously, in a total volume of 0.5 mL/mouse) was administered immediately after CLP as part of the resuscitation fluid. Mice were monitored for survival and sepsis-associated clinical signs. Disease severity was scoring done on days 0, 3, 4, 5, and 6 post-CLP using the murine sepsis score (MSS) (Shrum et al. 2014).

### Statistical analysis

All statistical tests were performed with GraphPad Prism 9 software (GraphPad, La Jolla, CA). Values are presented as individual samples and or mean ± SEM. Statistical analysis of mean differences between groups was performed using two-way ANOVA, paired t-test, Student’s t-test, Welch’s t-test, Mann–Whitney U-test, and mixed effects analysis with multiple comparisons as indicated in respective results. For all analyses, P ≤ 0.05 was considered statistically significant.

## Results

### IL-1β signals through neuronal TRPA1 to activate hypothermia

**Fig. 1:**
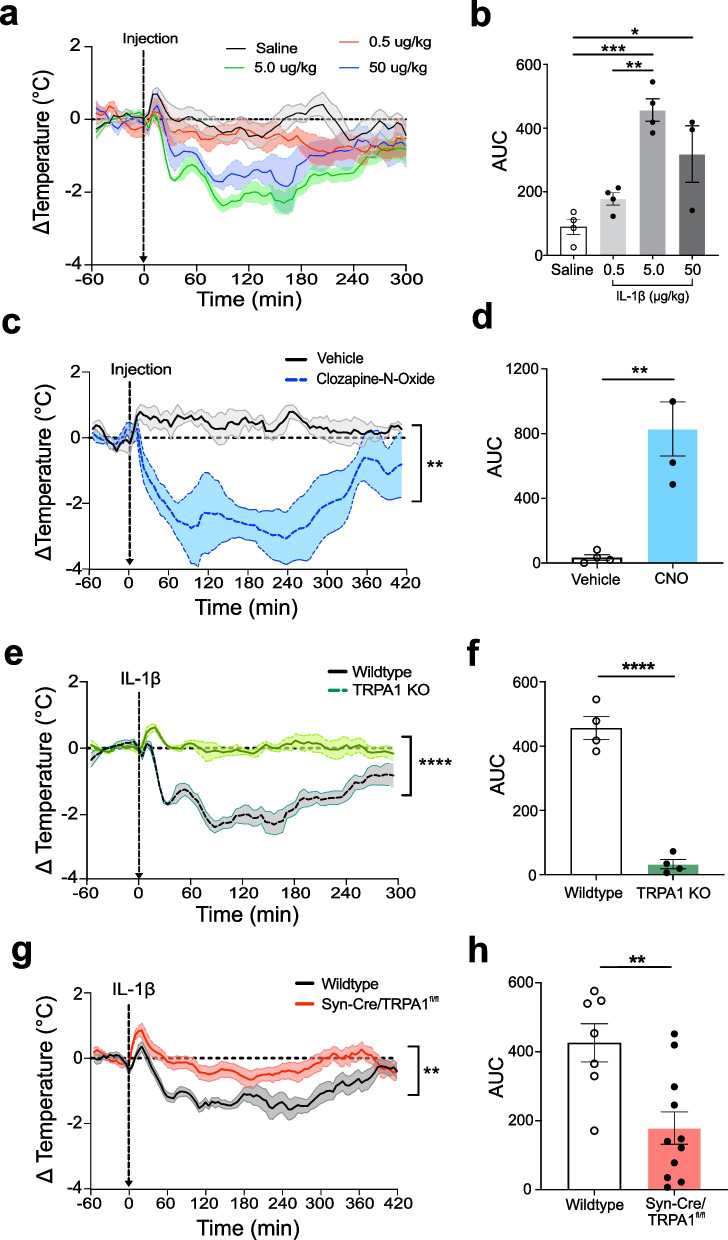
Neuronal TRPA1 is required for IL-1β-mediated thermoregulation. **a** Changes in core body temperature in wildtype (B6.129PF) mice in response to i.p. injection of saline (n = 4), 0.5 µg/kg of IL-1β (n = 4), 5 µg/kg of IL-1β (n = 4) and 50 µg/kg of IL-1β (n = 3). The dotted line represents the baseline. Data is averaged over 5-min increments and presented as mean ± SEM (solid line with shaded area). **b** Area under the curve (AUC) analysis of data in **a** with respect to a baseline of y = 2. Data presented as individual mouse datapoints with mean ± SEM. One-way ANOVA with Tukey multiple comparison; **p < 0.01, ***p < 0.001. **c** Changes in core body temperature in TRPA1-Cre/Gq-DREADD mice after i.p. injection (dashed arrow, time 0) of CNO (5 mg/kg) or vehicle (n = 4/group). Two-way RM-ANOVA; ***p < 0.001. **d** AUC analysis of data in **c**. Individual mouse datapoints with mean ± SEM presented. Unpaired t-test with Welch’s correction; **p < 0.01. **e** Changes in core body temperature in wildtype (B6.129PF, n = 4) and TRPA1 KO mice (n = 4) after i.p. administration (dashed arrow, time 0) of IL-1β (5 µg/kg). Data is averaged over 5-min increments and presented as mean ± SEM. 2-way RM-ANOVA; ****p < 0.0001. **f** AUC analysis of data in **e**. Data presented as individual mouse datapoints with mean ± SEM. Unpaired t-test with Welch’s correction; ****p < 0.0001*.* **g** Changes in core body temperature in wildtype (C57 BL/6, n = 7) and Syn-Cre/TRPA1^fl/fl^ mice (n = 11) after administration (dashed arrow, time 0) of IL-1β (5 µg/kg). Data was averaged over 5-min increments and presented as mean ± SEM. Two-way RM-ANOVA; **p < 0.01. **h** AUC analysis comparing wildtype and Syn-Cre/TRPA1^fl/fl^ mice with respect to a baseline of y = 2. Data presented as individual mouse datapoints with mean ± SEM. Unpaired t-test with Welch’s correction; **p < 0.01

Administration of strongly pro-inflammatory agents to rodents elicit changes in body core temperature which is a major feature of the rodent sickness response to injury and infection. Rodent thermoregulatory responses to cytokines or endotoxin depend upon both dose and ambient temperature, with higher doses administered at temperatures below the thermal neutral point eliciting hypothermia (Leon 2004). To assess for a potential role of TRPA1 nociceptors in IL-1β-mediated hypothermia, we implanted telemetry sensors into the abdominal cavity of mice to monitor core body temperature in real time. In experiments performed below the thermoneutral setpoint, intraperitoneal (i.p.) administration of vehicle (saline, 8.0 µL/kg) or low dose IL-1β (0.5 µg/kg) fails to induce significant changes in body temperature (Fig. 1a, b). In contrast, administration of higher doses of IL-1β (5 µg/kg or 50 µg/kg i.p.) induces hypothermia. Because we observed that 5 µg IL-1β /kg elicited maximum change in core temperature, this dose was administered for all subsequent experiments.

Prior work has reported that TRPA1 agonists stimulate hypothermia (Matsuo et al. 2021a; Gentry et al. 2015). To confirm this observation, we generated transgenic TRPA1-Cre/Gq-DREADD mice expressing Gq-DREADD (Gq-coupled Designer Receptor Exclusively Activated by Designer Drug) under the control of the TRPA1-Cre allele. Gq-DREADD receptors are muscarinic G-protein-coupled receptors (hM3Dq) modified to be activated exclusively by clozapine N-oxide (CNO) (an inert and inactive clozapine metabolite) and not by endogenous neurotransmitters (Roth 2016). Intraperitoneal administration of CNO to TRPA1-Cre/Gq-DREADD mice induces a significant decrease in core body temperature reaching a nadir (~ 3 °C drop) within 2 h; this is not observed in vehicle-administered controls (Fig. 1c, d). Because back metabolism of CNO can produce the parent, biologically active compound clozapine in rodents (Martinez et al. 2019), we performed additional controls by administering CNO to normal animals. The results show no effect of CNO on core body temperature (Additional file 1: Fig. S1a).

Next, to determine whether TRPA1 is required for IL-1β-activated hypothermia, we monitored body temperature in TRPA1 knock out (KO) mice receiving IL-1β. As expected, IL-1β induces hypothermia in wildtype control mice. However, administration of IL-1β fails to mediate hypothermia in TRPA1 KO mice (Fig. 1e, f). To exclude the possibility that TRPA1 KO mice are incapable of developing hypothermia, we next administered an independent hypothermia-inducing cytokine to TRPA1 KO mice. As expected, TNF administration to WT mice induces significant hypothermia (Schieber and Ayres 2016). However, in contrast to IL-1β, TNF (0.4 mg/kg) administered to TRPA1 KO mice also induces a rapid decrease in core body temperature (Additional file 1: Fig. S1b). Together these findings indicate TRPA1 is required for the hypothermia response mediated by IL-1β, but not TNF.

Because TRPA1 is expressed by non-neuronal cells types, including monocytes and macrophages (Fernandes et al. 2012), we next sought to selectively ablate TRPA1 from neurons and reassess the hypothermia responses to IL-1β. TRPA1 expression was ablated specifically in neurons by crossing synapsin-Cre (Syn-Cre) mice with floxed TRPA1 mice (TRPA1^f/f^) to produce Syn-Cre/TRPA1^fl/fl^ mice (Additional file 1: Fig. S2a) (Camilli et al. 1990; Greengard et al. 1993). Although IL-1β (5 µg/kg, i.p.) significantly decreases body temperature in wildtype controls, it fails to induce hypothermia in Syn-Cre/TRPA1^fl/fl^ mice (Fig. 1g, h). This finding provides direct evidence that TRPA1 positive neurons are required to mediate the hypothermia response to IL-1β.

### Nodose ganglion nociceptors co-express interleukin 1 receptor and TRPA1

**Fig. 2:**
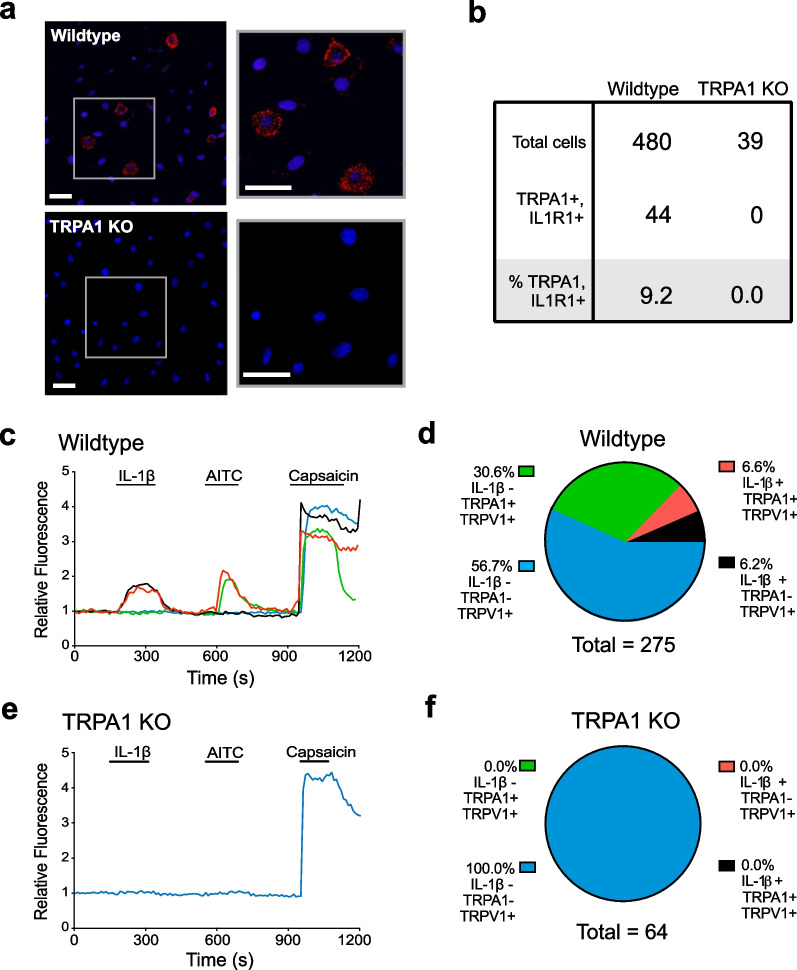
TRPA1 and IL1R1 co-localize on nodose ganglia sensory neurons, and TRPA1 is required for IL-1β mediated calcium signaling. **a** Representative in situ image of proximity ligation assay showing close proximity of TRPA1 and IL1R1 on nodose ganglion neurons (Scale bar = 50 µm). Nodose sensory neurons were isolated from wildtype and TRPA1 KO mice and subjected to in situ proximity ligation assay (“Methods”), using primary antibody pairs of anti-TRPA1 and anti-IL1R1 antibodies. The cells were counterstained with DAPI (blue) to visualize the nucleus. **b** Quantification of nodose ganglion neurons showing TRPA1 and IL1R1 co-localization. **c**–**f** IL-1β-induces sensory neuron activation in a TRPA1-dependent manner. Nodose ganglion sensory neurons harvested from **c** wildtype (B6.129PF) or **e** TRPA1 KO mice were stimulated in culture with IL-1β (20 µg/mL), AITC (100 µM; TRPA1-agonist) and capsaicin (10 µM; TRPV1-agonist) indicated by the labeled black line segments. **c**, **d** Sensory neurons from wildtype (B6.129PF) mice respond to IL-1β. **c** Representative examples of calcium traces of wildtype (B6.129PF) sensory neurons responding to IL-1β, AITC, and capsaicin (blue line); to IL-1β and capsaicin (red line); to AITC and capsaicin (green line) and only capsaicin (black line) are shown. **d** Percentage breakdown of wildtype calcium responses. Neurons responding to IL-1β, AITC, and capsaicin (blue); to IL-1β and capsaicin (red); AITC and capsaicin (green); and only capsaicin (yellow), are shown. These data were obtained from 17 coverslips cultured using nodose neurons harvested from 16 to 20 mice. **e**, **f** Sensory neurons from TRPA1 KO mice fail to respond to IL-1β. **e** Representative calcium traces of sensory neurons isolated from TRPA1 KO in response to IL-1β, AITC and capsaicin. **f** Percentage breakdown of TRPA1 KO calcium responses. Neurons responding to IL-1β, AITC, and capsaicin (blue); to IL-1β and capsaicin (red); AITC and capsaicin (green); and only capsaicin (yellow)

We and others have reported previously that neurons express IL-1β receptors, but whether TRPA1^+^ nociceptors express IL-1β receptors was previously unknown. Accordingly, a proximity ligation assay (PLA) was utilized to assess receptor co-expression on vagus neurons isolated from nodose ganglia, the anatomic site where the cell bodies of vagus sensory afferent neurons reside. PLA utilizes paired antibody-oligonucleotide conjugates which can be amplified by PCR reaction only if the protein targets of the antibodies are < 40 nm apart. Using antibodies specific to IL1R1 and TRPA1, PLA analysis of confocal fluorescence microscopy revealed that IL1R1 and TRPA1 co-localize on nodose ganglia neurons isolated from wildtype mice (Fig. 2a, upper panels: red cells). In contrast, we failed to observe co-localizing fluorescence on nodose ganglia neurons isolated from TRPA1 KO mice (Fig. 2a, lower panels). Quantification of 480 wildtype nodose neurons reveal that 9% co-expressed TRPA1 and IL1R1 (Fig. 2b).

### TRPA1 is required for IL-1β-mediated calcium signaling in nodose ganglion sensory neurons

To investigate whether TRPA1 is required for IL-1β-mediated vagus nociceptor stimulation, Fluo-4 based in vitro calcium imaging was applied to dissociated nodose ganglion sensory neurons harvested from wildtype and TRPA1 KO mice. Application of IL-1β induces calcium flux in a subset of nodose neurons which also respond to allyl isothiocyanate (AITC), a TRPA1 ligand (Hinman et al. 2006), and to capsaicin, a TRPV1 agonist (Caterina et al. 1997) (Fig. 2c, d). Increases in florescent intensity are observed in wildtype nodose neurons which selectively respond to IL-1β, AITC (TRPA1 positive) and capsaicin (TRPV1 positive) (Fig. 2c). Of 275 neurons studied, 6.6% are IL-1β and TRPA1 responsive (Fig. 2d). In contrast, nodose ganglion neurons harvested from TRPA1 KO mice fail to respond to AITC and IL-1β (Fig. 2e, f), providing direct evidence that TRPA1 is required for IL-1β-mediated neuronal activation.

**Fig. 3:**
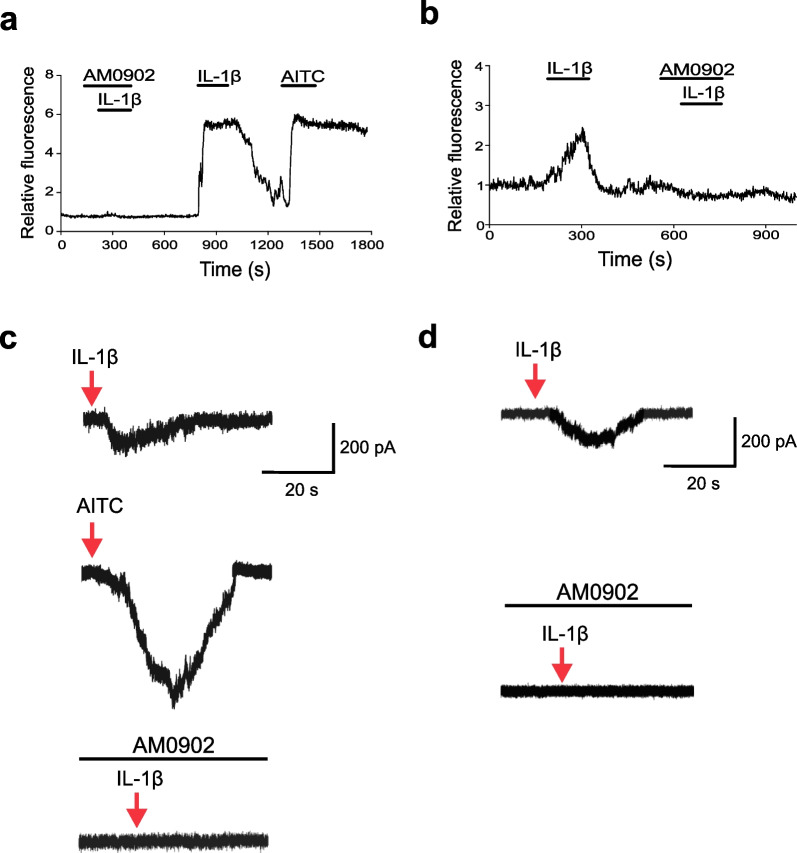
TRPA1 antagonist blocks IL-1β induced calcium flux and current in nodose ganglion sensory neurons. **a**, **b** Nodose ganglion sensory neurons harvested from VGlut2-cre/GCaMP3 mice were stimulated with IL-1β (20 µg/mL), AITC (100 µM; TRPA1-agonist) and AM0902 (10 µM; TRPA1-antagonist) indicated by the labeled black lines. **a** Representative calcium traces of sensory neurons. Nodose ganglia neurons fail to respond to IL-1β in the presence of TRPA1-antagonist AM0902. Washing of AM0902 restores sensory neuron responses to both IL-1β and TRPA1-agonist AITC. **b** Representative calcium traces of sensory neurons responding to IL-1β. Independent of the timing of IL-1β administration, addition of TRPA1-antagonist AM0902 to the culture during blocks IL-1β-induced calcium flux. **c**, **d** IL-1β evokes current in TRPA1^+^ sensory neurons. Nodose ganglion sensory neurons harvested from VGlut2-Cre/GCaMP3 mice were first exposed to IL-1β. Florescent imaging was carried out on the microscope to identify IL-1β-responsive neurons in the nodose culture. The cells were washed, and IL-1β-responsive neurons were selectively recorded using patch-clamp and responses to a TRPA1 agonist and antagonist sequentially recorded in individual neurons. **c** Representative response to IL-1β and TRPA1-agonist AITC. When held at − 70 mV, IL-1β (20 µg/mL) and AITC (100 µM) induced inward currents (arrows), AITC inward currents were blocked by TRPA1 antagonist AM0902 (50 µM). **d** IL-1β-induced responses were blocked by TRPA1-antagonist. Representative response to IL-1β monitored in a patch-clamp recording of nodose ganglion neurons. When held at − 70 mV, IL-1β (20 µg/mL) induced inward currents (arrow) were blocked by bath application of TRPA1 antagonist AM0902 (50 µM)

Next, we selectively inhibited TRPA1 signaling to assess whether this strategy also inhibits IL-1β-induced neuronal activation using the potent, selective TRPA1 antagonist AM0902 (which has an IC_50_ of 0.02 µM and does not inhibit TRPV1) (Schenkel et al. 2016). To do this using calcium imaging technologies, we first generated VGlut2-Cre/GCaMP3 mice which express genetically encoded calcium indicator 3 (GCaMP3) in sensory neurons directed by the vesicular glutamate transporter type 2 (VGlut2) promoter, because VGlut2 is expressed in glutamatergic sensory neurons (Brumovsky et al. 2007). GCaMP3 is specifically expressed in > 99% of the nodose ganglion neurons isolated from VGlut2-Cre/GCaMP3 mice (Chang et al. 2015; Corbett et al. 2005). When these neurons were exposed to IL-1β in the presence of AM0902, TRPA1 inhibition by AM0902 abolishes IL-1β-induced calcium influx (Fig. 3a). Moreover, washout of AM0902 followed by reapplication of IL-1β or AITC to the neurons produced corresponding increases in calcium influx (Fig. 3a). Blocking the TRPA1 channel using AM0902 following an initial IL-1β exposure also leads to inhibition of IL-1β-mediated calcium flux (Fig. 3b). Finally, stimulation of nodose ganglia sensory neurons from VGlut2-Cre/GCaMP3 mice, either in vitro or ex vivo using intact nodose ganglia preparations, with another TRPA1 agonist, polygodial, show a similar activation profile (Additional file 1: Figs. S3, S4), characterized by ~ 4% of neurons responding both to polygodial and to IL-1β (Additional file 1: Table S1).

Whole cell patch-clamp recordings of nodose ganglion sensory neurons exposed to IL-1β was performed using calcium transient analysis. With the neuron voltage clamped at − 70 mV, application of IL-1β (20 µg/mL) stimulated slow inward currents in 4 out of 332 (1.2%) nodose ganglion sensory neurons in culture (Fig. 3c). The mean amplitude of IL-1β-induced currents is 108 ± 23 pA (n = 4). As predicted from earlier results, all IL-1β responsive nodose ganglion nociceptors also responded to AITC (100 μM; Fig. 3c). AITC induces peak inward currents (410 ± 39 pA, n = 4) 3.8-fold greater than that elicited by IL-1β (Fig. 3c). In agreement with the calcium influx experiments, application of the TRPA1-antagonist AM0902 (50 µM), blocks the activating effects of IL-1β (n = 3; Fig. 3c, d). In current-clamp mode, the threshold of action potentials (− 25 ± 2 mV) induced by current injections (100 pA, 200 ms) is reduced by IL-1β application to − 31 ± 3 mV (n = 4), but is reversed fully within 30 min after wash (data not shown). Together these calcium flux imaging and patch-clamp recording results indicate that TRPA1 is required for IL-1β-induced vagus nociceptor activation.

### IL-1β administration increases vagus nerve electrical activity in a TRPA1-dependent manner

**Fig. 4:**
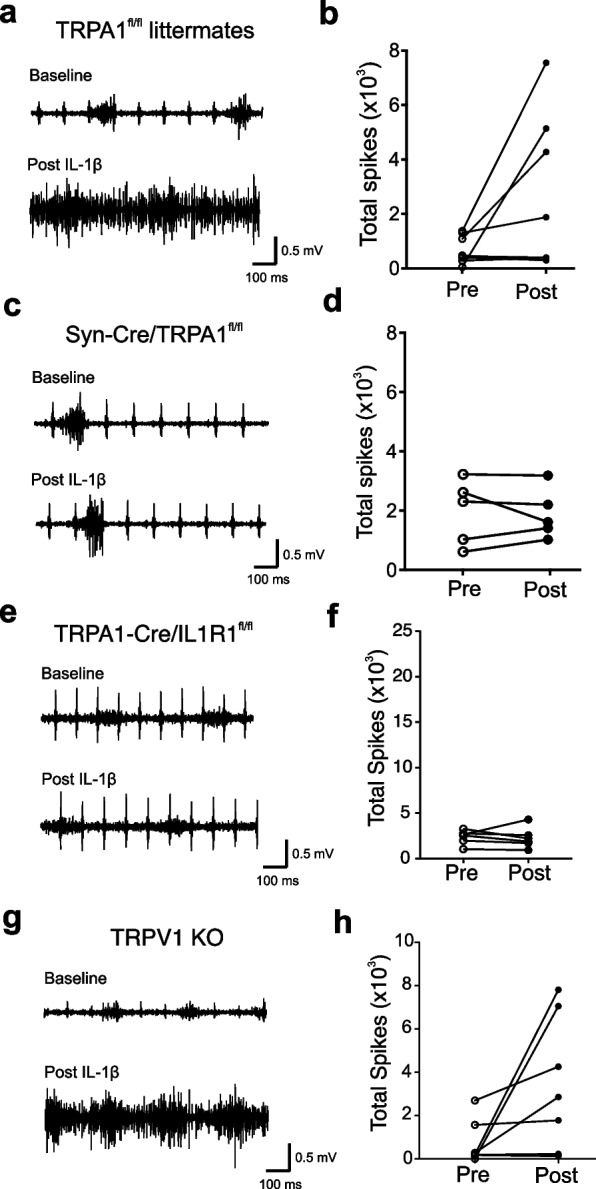
IL-1β induced vagus nerve electrical activity requires TRPA1 and IL1R1 but not TRPV1. **a**, **c** Representative recordings of the vagus nerve signals shown for **a** TRPA1^fl/fl^ littermate control mice and **c** Syn-Cre/TRPA1^fl/fl^ mice starting before (baseline) and after IL-1β administration (350 ng/kg, post-IL-1β). Examples of the baseline and post-IL-1β administration responses are shown. Data is representative of 5–8 animals per group. **b**, **d** Total spike count during recordings over the entire 5-min pre- and 5-min post-IL-1β administration in **b** TRPA1^fl/fl^ littermate control mice (n = 8) and **d** Syn-Cre/TRPA1^fl/fl^ mice (n = 5). Data is presented as individual mouse data point. **e**, **g** Representative recordings of the vagus nerve signals shown for **e** TRPA1-Cre/IL1R1^fl/fl^ mice and **g** TRPV1 KO mice starting before (baseline) and after IL-1β administration (350 ng/kg, Post-IL-1β). Data is representative of 6–7 animals per group. **f**, **h** Total spike count over the entire 5-min pre- and 5-min post-IL-1β administration recordings in TRPA1-Cre/IL-1R1^fl/fl^ mice (n = 6) and TRPV1 KO mice (n = 7). Data are represented as individual mouse data points

It has been previously established that IL-1β induces neuronal activation through calcium influx and induction of action potentials (Steinberg et al. 2016; Zanos et al. 2018; Binshtok et al. 2008; Miller et al. 2009). To assess directly the role of IL-1β and vagus nerve signaling via TRPA1, a bipolar cuff electrode was placed on the cervical vagus nerve in anesthetized control and TRPA1 KO mice as previously described (Steinberg et al. 2016; Zanos et al. 2018). As expected from the results of prior work, IL-1β induces significant increases in vagus nerve activity in control mice (Additional file 1: Fig. S5a, b). However, IL-1β fails to induce vagus nerve activity in TRPA1 KO mice (Additional file 1: Fig. S5c, d). In other studies, administration of IL-1β to Syn-Cre/TRPA1^fl/fl^ mice and TRPA1^fl/fl^ littermate controls induced significant increases in vagus nerve activity only in the TRPA1^fl/fl^ littermate controls (Fig. 4a, b), but not in the Syn-Cre/TRPA1^fl/fl^ mice (Fig. 4c, d). The vagus nerve responses to IL-1β are also abolished in the TRPA1-Cre/IL1R1^fl/fl^ mice derived by crossing TRPA1-Cre mice with floxed IL1R1 mice (IL1R1^f/f^) (Fig. 4e, f). Because the majority of TRPA1 positive neurons also express TRPV1, we also assessed these responses to IL-1β in TRPV1 ablated mice (Fig. 4g, h), providing additional evidence that neuronal TRPA1 and IL1R1 are specifically required to mediate IL-1β-induced electrical signaling and hypothermia.

### Activation of vagus nerve TRPA1-signaling inhibits pro-inflammatory cytokine production in endotoxemia

**Fig. 5:**
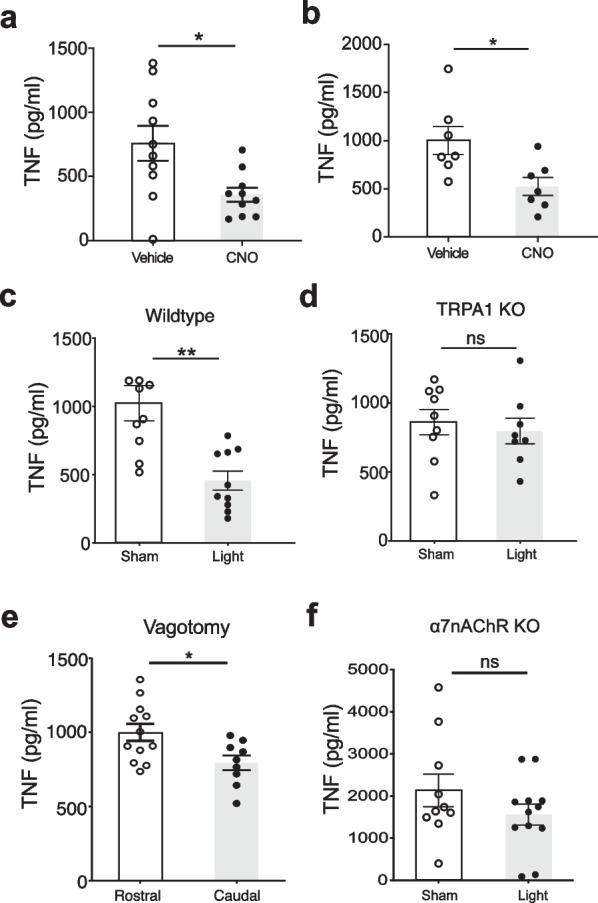
Activation of vagus nerve TRPA1 signaling inhibits TNF during endotoxemia. **a** TRPA1-Cre/Gq-DREADD mice were injected with either CNO (5 mg/kg) or vehicle i.p. (n = 10/ group) 1 h prior to LPS (0.3 mg/kg, i.p.). For all endotoxemia experiments, serum TNF levels were analyzed at 90 min post-LPS. Individual mouse data points with mean ± SEM are presented. Unpaired t-test with Welch’s correction; *p < 0.05. **b** Vehicle or 10 µL CNO (0.25 mg/kg) was administered directly to the exposed vagus nerve (n = 7 per group) in TRPA1-Cre/Gq-DREADD mice 2 h prior to LPS (1.0 mg/kg). Individual mouse data points with mean ± SEM are presented. Unpaired t-test with Welch’s correction; *p < 0.05. Optovin was directly applied to the vagus nerve and exposed to sham or 405 nm light stimulation (1000 mA, 10 Hz, with a 10% duty cycle for 5 min) 2 h prior to LPS injection. **c** Wildtype mice (B6.129PF) receiving sham (n = 9) or light (n = 10) stimulation. Unpaired t-test with Welch’s correction; **p < 0.01. **d** TRPA1 KO mice receiving sham (n = 9) or light (n = 8) stimulation. Individual mouse data points with mean ± SEM are presented. Unpaired t-test with Welch’s correction; ns: not significant. **e** Vagotomy was performed either rostral (efferent; n = 12) or caudal (afferent; n = 9) with respect to optovin administration site in wildtype mice (B6.129PF) and subjected to sham or 405 nm light stimulation 2 h prior to endotoxin injection. Individual mouse data points with mean ± SEM are presented. Unpaired t-test with Welch’s correction; *p < 0.05. **f** Vagus nerve was exposed in *α7nAChR* KO mice and optovin was directly applied, and subjected to 405 nm light (n = 12) or sham stimulation (n = 10) 2 h prior to LPS injection. Individual mouse data points with mean ± SEM are presented. Unpaired t-test with Welch’s correction; ns: not significant

While these results indicate vagus TRPA1 nociceptors mediate hypothermia and activate vagus nerve signaling, it remained unknown whether TRPA1 nociceptors activated by IL-1β also stimulate a reflex anti-inflammatory response to inhibit cytokine production and inflammation. To study this, we produced mice expressing DREADD (Gq-DREADD) in TRPA1 positive nociceptors. These TRPA1-Cre/Gq-DREADD mice received bacterial endotoxin (lipopolysaccharide, LPS) to stimulate cytokine storm while also undergoing selective activation of TRPA1 positive nociceptors using CNO administered either systemically (Fig. 5a) or directly to the vagus nerve (Fig. 5b). CNO delivered by either route significantly inhibited serum TNF levels following LPS, consistent with activation of the protective reflex anti-inflammatory response. We then used optopharmacology to activate TRPA1 nociceptors on the vagus nerve in vivo. Optovin, a photoactivated TRPA1 ligand (Kokel et al. 2013), was applied directly onto the cervical vagus nerve and then triggered by exposure to light (Additional file 1: Fig. S6). This significantly inhibits TNF production in mice receiving LPS to stimulate cytokine storm, a classic assay of reflex anti-inflammatory activity (Fig. 5c). Application of optovin to the cervical vagus nerve of TRPA1 KO mice in vivo fails to inhibit TNF production (Fig. 5d). A similar result is observed using polygodial, which also inhibits TNF production in wildtype mice, but not in TRPA1 KO mice (Additional file 1: Fig. S7a, b). Thus, independent and specific TRPA1 agonists each stimulate a reflex anti-inflammatory response which in turn inhibits cytokine storm induced by LPS.

To determine the role of afferent and efferent vagus nerve signaling in these experiments we next performed selective vagotomy by transecting the left cervical vagus nerve rostral or caudal to the optically-stimulated area. Eliminating afferent vagus nerve signals to the brainstem, while maintaining efferent vagus signals to the spleen, significantly abolishes TRPA1-dependent inhibition of TNF (rostral; Fig. 5e). In contrast, vagotomy caudal to the stimulation site which maintains afferent signaling preserves optovin-mediated anti-inflammatory activity (caudal; Fig. 5e). This can be mediated by efferent outflow in the intact contralateral vagus and from other neuroimmune circuits stimulating sympathetic outflow (Tanaka 2021). Because the inflammatory reflex inhibits cytokine storm by signaling via α7nAChR (Wang et al. 2003), we also subjected α7nAChR KO mice with intact afferent vagus nerve fibers to optovin photostimulation. This fails to activate reflex anti-inflammatory activity in these animals, because TNF is not inhibited during endotoxemia (Fig. 5f). Together, these data establish that vagus TRPA1^+^ nociceptors are necessary and sufficient to stimulate a reflex anti-inflammatory response via afferent vagus nerve fiber signaling and inhibit TNF production during endotoxemia.

### Protective role of TRPA1 activation in a model of bacterial sepsis

**Fig. 6:**
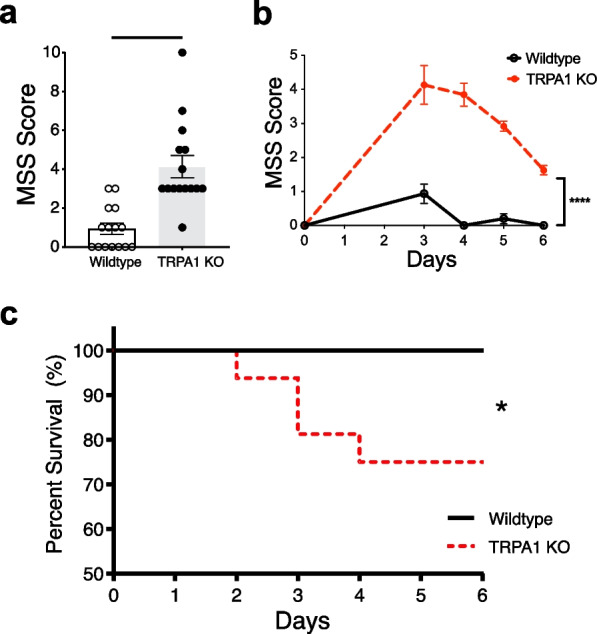
TRPA1 is required to improve survival in experimental sepsis. **a**–**c** Wild type (B6.129PF) and TRPA1 KO mice were subjected to cecal ligation and puncture (CLP) procedure. **a** Murine Sepsis Score (MSS) assessed in wildtype (B6.129PF; n = 15) and TRPA1 KO mice (n = 12–16) on day 3 post-CLP. Data is presented as individual mouse data points with mean ± SEM. Unpaired t-test with Welch’s correction. ****p < 0.0001. **b** Murine sepsis scores assessed in wildtype (B6.129PF; n = 15) and TRPA1 KO mice (n = 12–16) on pre-CLP (Day 0) and post-CLP days (day 3, 4, 5, and 6). Data is presented as mean ± SEM. Mixed effects analysis; ****p < 0.0001. **c** Sepsis mortality rate in wildtype (B6.129PF; n = 15) and TRPA1 KO mice (n = 16) subjected to sublethal CLP. Sublethal CLP protects wildtype mice with no mortality, but induces 25% mortality in TRPA1 KO mice by day 4. Log-rank (Mantel–Cox) test, *p < 0.05

Because TRPA1 is required to activate the homeostatic, protective reflex anti-inflammatory activity, we reasoned that TRPA1 deficiency renders animals more sensitive to sepsis. Wildtype and TRPA KO mice subjected to cecal ligation and puncture (CLP)-induced polymicrobial sepsis developed clinical signs of sickness syndrome which is readily scored and stratified. The sepsis severity score in TRPA1 KO mice is significantly increased as compared to WT control mice for at least 6 days (Fig. 6a, b) and moreover, mortality is increased significantly in TRPA1 KO mice as compared to WT (p < 0.05; Fig. 6c).

## Discussion

The results of these experiments show that IL-1β stimulates TRPA1 afferent vagus nociceptors to activate an anti-inflammatory response which is protective against otherwise lethal cytokine-mediated inflammation and sepsis. Additionally, direct activation of TRPA1 using optopharmacology and chemogenetic methods attenuates TNF production, whereas deletion of TRPA1 or interrupting afferent vagus nerve signaling results in a loss of these anti-inflammatory effects and decreases survival from infection. These findings reveal that the role of IL-1β in host defense is complex and pleiotropic because it is both a pro-inflammatory mediator capable of causing tissue injury, and an anti-inflammatory signal via vagus TRPA1-dependent suppression of pro-inflammatory cytokine production which reduces collateral damage. Similarly, abundant prior work has implicated a key role of TRPA1 in inflammation, pain, itch, and as a sensor of inflammatory mediators, cellular stress, and tissue damage (Bautista et al. 2013; Viana 2016; Silverman et al. 2020; Landini, et al. 2022). The present data now reveal the role of TRPA1 as also pleiotropic in mediating protective, homeostatic mechanisms in inflammation and infection because the results indicate an important role for vagus nerve TRPA1 ion channels in activating the thermoregulatory responses to IL-1β. As shown here, TNF administration, but not IL-1β, elicits hypothermia in the TRPA1 ablated animals, which means that IL-1β-induced hypothermia is specific to vagus TRPA1 nociceptors and that other as yet unknown neural circuits mediate the hypothermic response to TNF.

The proximity ligation assay (PLA) results indicate IL-1R1 and TRPA1 are co-expressed and in close proximity on the cell surface in a subset of afferent vagus nerve fibers, supporting the possibility that the cytokine receptor regulates or induces ion channel flux and neuronal signaling. However, several caveats concerning this technique require consideration. First, the specificity of commercially available anti-TRPA1 antibodies has been questioned [e.g., Virk et al. (2019)] and strict specificity of the polyclonal antibody we used has not been confirmed by published data. However, the loss of responses observed in animals with TRPA1 specifically knocked out of vagus neurons indicates that the positive signal requires TRPA1. An additional caveat is that although the PLA findings are consistent with close physical proximity of these receptor and ion channel proteins, the technique cannot reliably confirm physical linkage (Alsemarz et al. 2018). Both IL-1R1 (Oakley et al. 2009) and TRPA1 (Startek 2019) translocate into lipid rafts within the cell membrane, localized regions of high lipid content in which membrane proteins are highly enriched, leading to an increased probability of close enough proximity which will generate a positive PLA result. Actual physical linkage of IL1R1 and TRPA1 will require additional confirming techniques or co-immunoprecipitation.

Details of the molecular mechanism by which IL-1β signaling through TRPA1 in vagus nerve sensory neurons remain to be clarified. Although the results of prior study have established that nociceptor neurons respond rapidly to IL-1β through activation of p38 mitogen-activated protein kinase (MAPK) resulting in an increase in action potential frequency and augmented pain behavior (Binshtok et al. 2008), the intervening molecular pathways linking receptor activation to physiological response also remain elusive. Additional insight obtained from studies of glomerular mesangial cells is potentially relevant, because IL-1β signaling via IL1R1 mediates TRPA1 ion channel Ca^2+^ influx which subsequently activates ERK MAPK-dependent cell proliferation (Soni et al. 2021). The signaling cascade which activates TRPA1 Ca^2+^ flux is initiated by an IL-1-dependent rapid increase in intracellular diacylglycerol (DAG) (Kester et al. 1989; Sedor et al. 1992). However, unlike a variety of cell membrane receptors, e.g., the bradykinin receptor 2 (Kadkova et al. 2017), DAG is not generated through protein kinase C (PKC), but rather by the action of phosphatidic acid phosphohydrolase (PAP), a lipid signaling pathway which provides important regulatory control of inflammatory signaling (Grkovich and Dennis 2009). The action of PAP does not generate inositol triphosphate and, therefore, does not directly result in the release of Ca^2+^ stored in the endoplasmic reticulum. Instead, the increase in intracellular Ca^2+^ depends upon extracellular Ca^2+^ flux through TRPA1 which activates cellular signaling (Soni et al. 2021). A similar pathway driving neuronal excitability could exist for neurons such that DAG interacts directly with TRPA1 (Soni et al. 2021) or through the activation of PKC, which could sensitize TRPA1 via the A kinase anchoring protein AKAP79/150 site present on the C terminal of TRPA1 (Kadkova et al. 2017) resulting in Ca^2+^ influx. Further study is required to evaluate the potential relevance of these molecular pathways in vagus nerve IL-1β/TRPA1 signaling.

Additional considerations are warranted concerning the role of hypothermia as part of a homeostatic, protective system. In mice a stereotypic “fear response” is induced by predator-specific odors, e.g., thiazolines, which trigger TRPA1 ion channels in sensory neurons, including vagus nociceptors (Matsuo et al. 2021a, b). As in the present study, the TRPA1 response activated by odorants is adaptive, promoting survival of stressors such as hypoxia by activating an energy conserving reflex consisting of immobility, hypothermia, bradycardia, and brain sparing hypometabolism, which also inhibits LPS-stimulated pro-inflammatory cytokines (Matsuo et al. 2021a). Mice also enter a hypometabolic state, termed torpor (Sunagawa and Takahashi 2016), when challenged by food scarcity, and torpor is also associated with hypothermia and hypometabolism. Thus, IL-1β, predator-associated odorant molecules, and food deprivation are examples of a stereotyped response to diverse stimuli which activate TRPA1 positive sensory neurons of the vagus to mount compensatory, protective responses. Although reflex anti-inflammatory responses have not been studied in fear responses and torpor, it is plausible to suggest this should be a fruitful line of investigation.

Patients with sepsis and other hyperinflammatory states (e.g., major burns or trauma) develop immune suppression with anergy to common antigens (Ward et al. 2008). This impaired immunity, termed “compensatory anti-inflammatory response syndrome”, persists following recovery from acute disease and is associated with recurrent infections and other complications (Delano and Ward 2016). Rodent survivors of CLP also have persistent immune suppression and constitutive increased signaling in the vagus nerve (Rana et al. 2018). Although a role for IL-1β has not been evaluated previously, the current study suggests that afferent TRPA1 vagus nociceptors may mediate this immunosuppression in response to IL-1β. From understanding mechanisms of vagus TRPA1 nociceptors and IL-1β in stimulating reflex anti-inflammatory responses may come novel therapeutic insights based on IL-1β-TRPA1 vagus nociceptor signaling.

## Conclusions

Here, we describe a previously unrecognized mechanism by which IL-1β activates afferent vagus nerve fibers to trigger hypothermia, a response which is abolished by selective silencing of neuronal TRPA1. Vagus nerve TRPA1 signaling also inhibits endotoxin-stimulated cytokine storm and significantly reduces the lethality of bacterial sepsis. Thus, IL-1β activates TRPA1 vagus nerve signaling in the afferent arm of a reflex anti-inflammatory response to inhibit cytokine release, induce hypothermia, and reduce the mortality of infection. This discovery establishes that TRPA1, an ion channel known previously as a pro-inflammatory detector of cold, pain, itch, and a wide variety of noxious molecules, also plays a specific anti-inflammatory role via nervous system regulation of anti-inflammatory responses.

## Supplementary Information

**Additional file 1: Figure S1.** **a** CNO administration does not cause hypothermia in normal mice. Changes in core body temperature in TRPA1-Cre mice after i.p. injection (dashed arrow, time 0) of CNO (5 mg/kg) or vehicle (n = 5/group). **b** TRPA1 is not required for TNF-induced hypothermia. Changes in core body temperature in wildtype (B6.129PF, n = 5) and TRPA1 KO mice (n = 10) after administration of TNF (0.04 mg/kg). Injection occurred at time 0 (dashed arrow). Data is averaged over 5-min increments and presented as mean ± SEM. Data presented as mean ± SEM. **Figure S2.** **a** Breeding strategy for the generation of Syn-Cre/TRPA1^fl/fl^ mice. **b** Breeding strategy for the generation of TRPA1/IL-1R^fl/fl^ mice. **Figure S3.** IL-1β induces activation of nodose sensory neurons responsive to polygodial and capsaicin. Representative GCaMP3 fluorescence signal in whole mount nodose ganglia from Vglut2-Cre/GCaMP3 mice, showing responses to IL-1β (200 µg/mL). Polygodial (200 µM) and capsaicin (10 µM) are applied to identify TRPA1- and TRPV1-expressing neurons, respectively. Examples of neurons responding to IL-1β, polygodial and capsaicin (black line), to polygodial and capsaicin (blue line), and only capsaicin (red line) are shown. **Figure S4.** IL-1β-induces sensory neuron activation in a TRPA1-dependent manner. **a** Representative GCaMP3 fluorescence signal in dissociated and cultured nodose ganglia neurons from Vglut2-Cre/GCamp3 mice showing responses to IL-1β (20 µg/mL) in the absence of TRPA1 antagonist AM0902 (10 µM). Addition of TRPA1-antagonist AM0902 inhibits IL-1β-induced responses. Polygodial (10 µM) is applied to identify TRPA1-expressing neurons. **b** Representative calcium traces of wildtype (B6.129PF; black solid line) and TRPA1 KO mice (red solid line) dissociated and cultured, nodose ganglia neurons showing responses to IL-1β (20 µg/mL), polygodial (10 µM; TRPA1-positive) and capsaicin (10 µM; TRPV1-positive) indicated by the labeled black lines. **Figure S5.** IL-1β fails to induce vagus nerve firing in TRPA1 KO mice. **a**, **c** Representative recordings of the vagus nerve signals shown for **a** wildtype mice and **c** TRPA1 KO mice before (baseline) and after IL-1β administration (350 ng/kg, Post-IL-1β). Data is representative of 6–7 animals per group. **b**, **d** Total spike count over the entire 5-min pre- and 5-min post-IL-1β administration recordings in **b** wildtype control mice (n = 6) and **d** TRPA1 KO mice (n = 7). Data is represented as individual mouse data points. **Figure S6.** Opto-pharmacological activation of TRPA1 on the vagus nerve. **a**–**c** Optovin was directly administered to the exposed cervical vagus in wildtype or TRPA1 KO mice nerve prior to stimulation with light. Animals were subjected to 405 nm light (1000 mA, 10 Hz, with a 10% duty cycle) stimulation or sham stimulation. **a** Optovin-induced activation of TRPA1 on cervical vagus nerve fibers. Representative neurogram recordings of wildtype mice without optovin, wildtype mice with optovin and TRPA1 KO mice with optovin. Horizontal blue bars indicate when the light pulse was on. Optovin + light stimulation induces compound action potentials in the vagus nerve in wildtype mice but not in TRPA1 KO mice. **b**, **c** Quantification of the amplitude of light evoked potentials. Amplitude of the optovin + light stimulation was quantitated in wildtype and TRPA1 KO mice. **b** Wildtype mice with light only (n = 6) or light with optovin (n = 6); **c** TRPA1 KO mice with light (n = 6) or light with optovin (n = 6). Data is presented as individual mouse data point with mean ± SEM. Paired t test, ***p < 0.001. **Figure S7.** TRPA1-specific agonist suppresses TNF production in endotoxemic wildtype mice but not in TRPA1 KO mice. Vehicle or TRPA1 agonist, polygodial (5 mg/kg, i.p.), was administered to **a** wildtype mice (B6.129PF, n = 5–6 per group) or **b** TRPA1 KO mice (n = 9–10 per group) 30 min prior to LPS administration (0.1 mg/kg, i.p.). Serum was obtained 90 min after endotoxin administration, and TNF was measured by ELISA. Data is presented as individual mouse data point with mean ± SEM. Unpaired t test with Welch’s correction; **p < 0.001. **Table S1.** IL-1β responsive sensory neuron population. Percentage of capsaicin responsive neurons that respond to polygodial, IL-1β, and both IL-1β and polygodial in intact and cultured nodose ganglion neurons isolated from Vglut2-Cre/GCaMP3, wildtype (B6.129PF), and TRPA1 KO mice.

## Data Availability

All data supporting the findings of this study are available within the paper and its additional materials. All study data are included in the article and its Additional file.
