# Supplementary material for: Transient Receptor Potential Ankyrin-1-expressing vagus nerve fibers mediate IL-1β induced hypothermia and reflex anti-inflammatory responses
Source: Mol Med. 2023 Jan 18;29:4. doi: 10.1186/s10020-022-00590-6 (PMC9847185; doi:10.1186/s10020-022-00590-6)
Supplement: Supplementary file 1 — Additional file 1: Figure S1. a CNO administration does not cause hypothermia in normal mice. Changes in core body temperature in TRPA1-Cre mice after i.p. injection (dashed arrow, time 0) of CNO (5 mg/kg) or vehicle (n = 5/group). b TRPA1 is not required for TNF-induced hypothermia. Changes in core body temperature in wildtype (B6.129PF, n = 5) and TRPA1 KO mice (n = 10) after administration of TNF (0.04 mg/kg). Injection occurred at time 0 (dashed arrow). Data is averaged over 5-min increments and presented as mean ± SEM. Data presented as mean ± SEM. Figure S2. a Breeding strategy for the generation of Syn-Cre/TRPA1fl/fl mice. b Breeding strategy for the generation of TRPA1/IL-1Rfl/fl mice. Figure S3. IL-1β induces activation of nodose sensory neurons responsive to polygodial and capsaicin. Representative GCaMP3 fluorescence signal in whole mount nodose ganglia from Vglut2-Cre/GCaMP3 mice, showing responses to IL-1β (200 µg/mL). Polygodial (200 µM) and capsaicin (10 µM) are applied to identify TRPA1- and TRPV1-expressing neurons, respectively. Examples of neurons responding to IL-1β, polygodial and capsaicin (black line), to polygodial and capsaicin (blue line), and only capsaicin (red line) are shown. Figure S4. IL-1β-induces sensory neuron activation in a TRPA1-dependent manner. a Representative GCaMP3 fluorescence signal in dissociated and cultured nodose ganglia neurons from Vglut2-Cre/GCamp3 mice showing responses to IL-1β (20 µg/mL) in the absence of TRPA1 antagonist AM0902 (10 µM). Addition of TRPA1-antagonist AM0902 inhibits IL-1β-induced responses. Polygodial (10 µM) is applied to identify TRPA1-expressing neurons. b Representative calcium traces of wildtype (B6.129PF; black solid line) and TRPA1 KO mice (red solid line) dissociated and cultured, nodose ganglia neurons showing responses to IL-1β (20 µg/mL), polygodial (10 µM; TRPA1-positive) and capsaicin (10 µM; TRPV1-positive) indicated by the labeled black lines. Figure S5. IL-1β fails to induce vagus [file 10020_2022_590_MOESM1_ESM.docx]

**Additional file 1 (Silverman et al):**

**
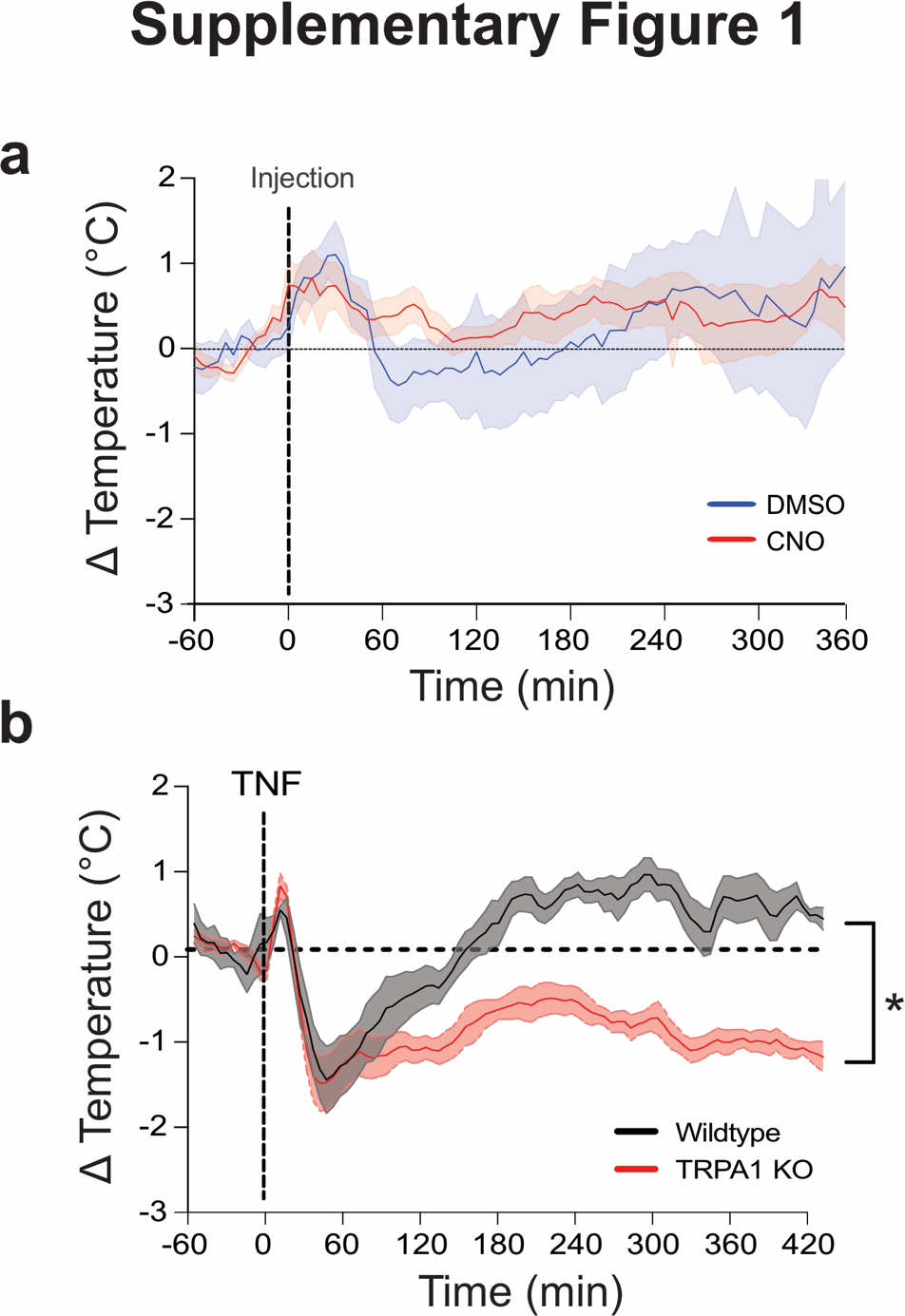
**

**Figure S1. (a)** CNO administration does not cause hypothermia in normal mice. Changes in core body temperature in TRPA1-Cre mice after i.p. injection (dashed arrow, time 0) of CNO (5 mg/kg) or vehicle (n=5/group). **(b)** TRPA1 is not required for TNF-induced hypothermia. Changes in core body temperature in wildtype (B6.129PF, n=5) and TRPA1 KO mice (n=10) after administration of TNF (0.04mg/kg). Injection occurred at time 0 (dashed arrow). Data is averaged over 5-minute increments and presented as mean ± SEM. Data presented as mean ± SEM.


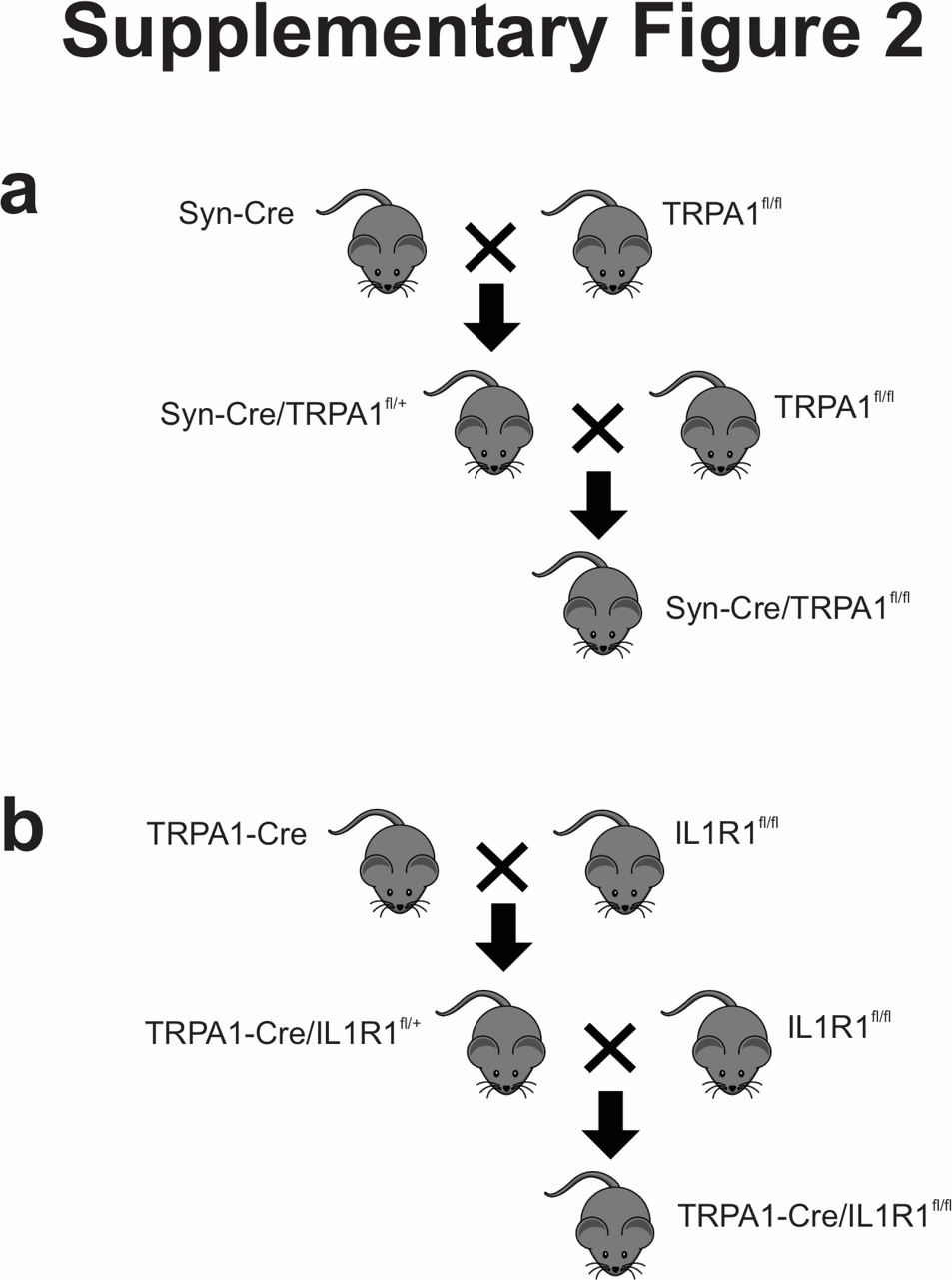


**Figure S2. (a)** Breeding strategy for the generation of Syn-Cre/TRPA1^fl/fl^ mice. **(b)** Breeding strategy for the generation of TRPA1/IL-1R^fl/fl^ mice.


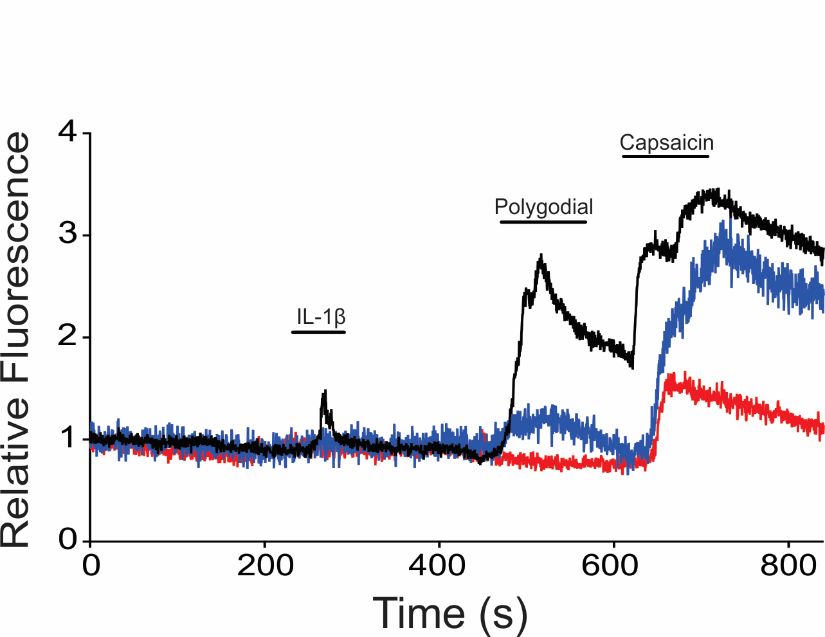


**Figure S3. IL-1β induces activation of nodose sensory neurons responsive to polygodial and capsaicin.** Representative GCaMP3 fluorescence signal in whole mount nodose ganglia from Vglut2-Cre/GCaMP3 mice, showing responses to IL-1β (200µg/ml). Polygodial (200µM) and capsaicin (10µM) are applied to identify TRPA1- and TRPV1-expressing neurons, respectively. Examples of neurons responding to IL-1β, polygodial and capsaicin (black line), to polygodial and capsaicin (blue line), and only capsaicin (red line) are shown.


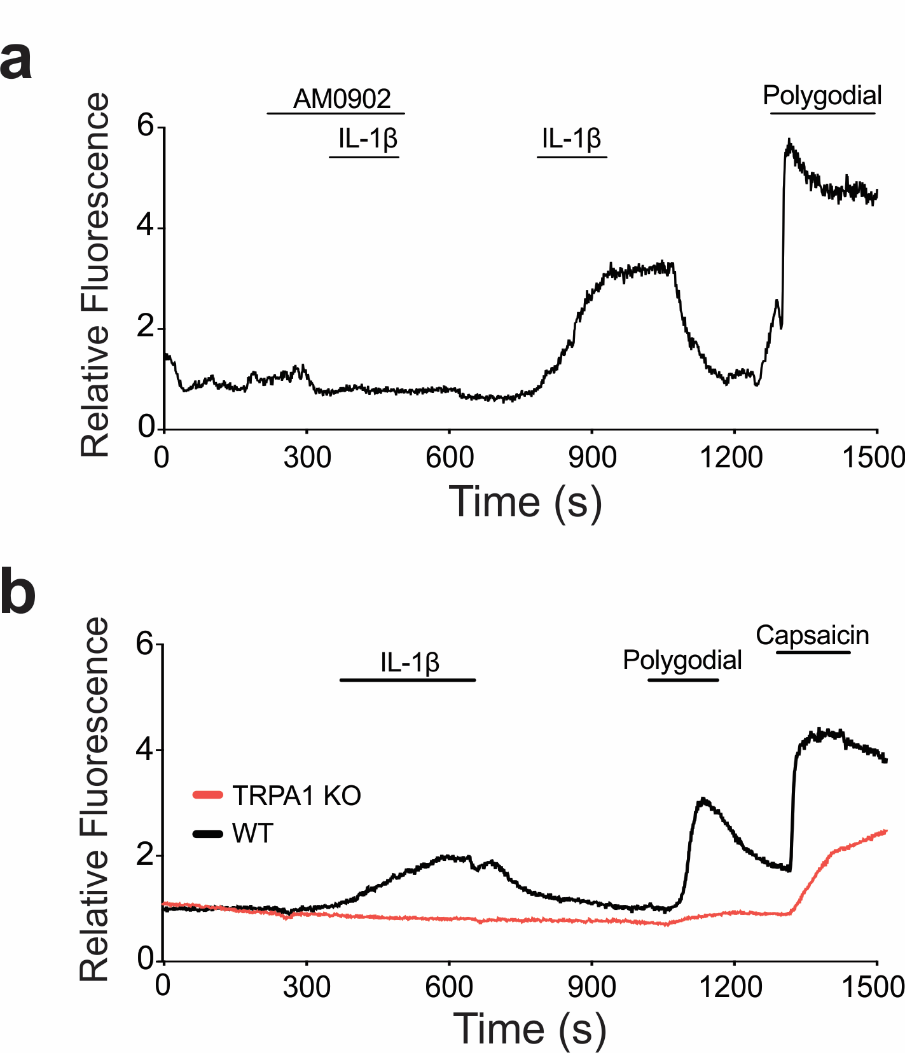


**Figure S4. IL-1β-induces sensory neuron activation in a TRPA1-dependent manner. (a)** Representative GCaMP3 fluorescence signal in dissociated and cultured nodose ganglia neurons from Vglut2-Cre/GCamp3 mice showing responses to IL-1β (20µg/ml) in the absence of TRPA1 antagonist AM0902 (10µM). Addition of TRPA1-antagonist AM0902 inhibits IL-1β-induced responses. Polygodial (10µM) is applied to identify TRPA1-expressing neurons. **(b)** Representative calcium traces of wildtype (B6.129PF; black solid line) and TRPA1 KO mice (red solid line) dissociated and cultured, nodose ganglia neurons showing responses to IL-1β (20µg/ml), polygodial (10µM; TRPA1-positive) and capsaicin (10µM; TRPV1-positive) indicated by the labeled black lines.

**
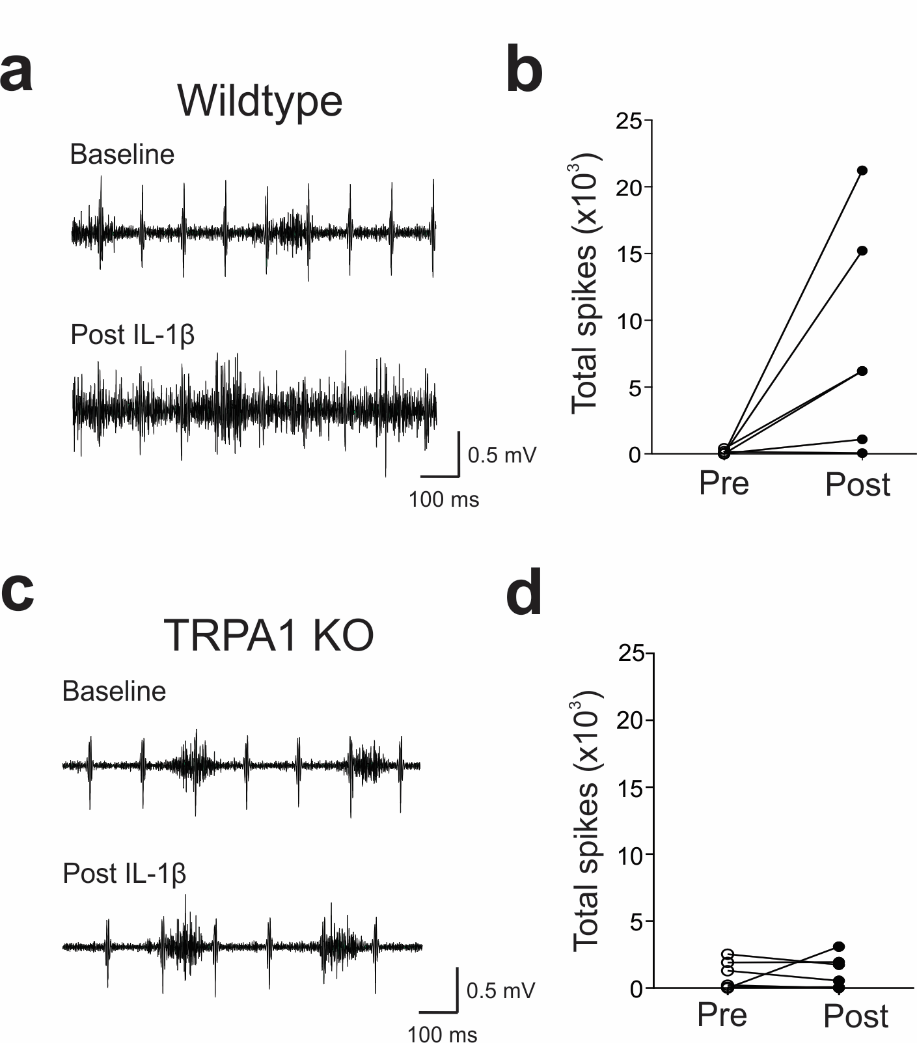
**

**Figure S5. IL-1β fails to induce vagus nerve firing in TRPA1 KO mice. (a, c)** Representative recordings of the vagus nerve signals shown for **(a)** wildtype mice and **(c)** TRPA1 KO mice before (baseline) and after IL-1β administration (350 ng/kg, Post-IL-1β). Data is representative of 6-7 animals per group. **(b, d)** Total spike count over the entire 5-minute pre- and 5-minute post- IL-1β administration recordings in **(b)** wildtype control mice (n = 6) and **(d)** TRPA1 KO mice (n = 7). Data is represented as individual mouse data points.

**
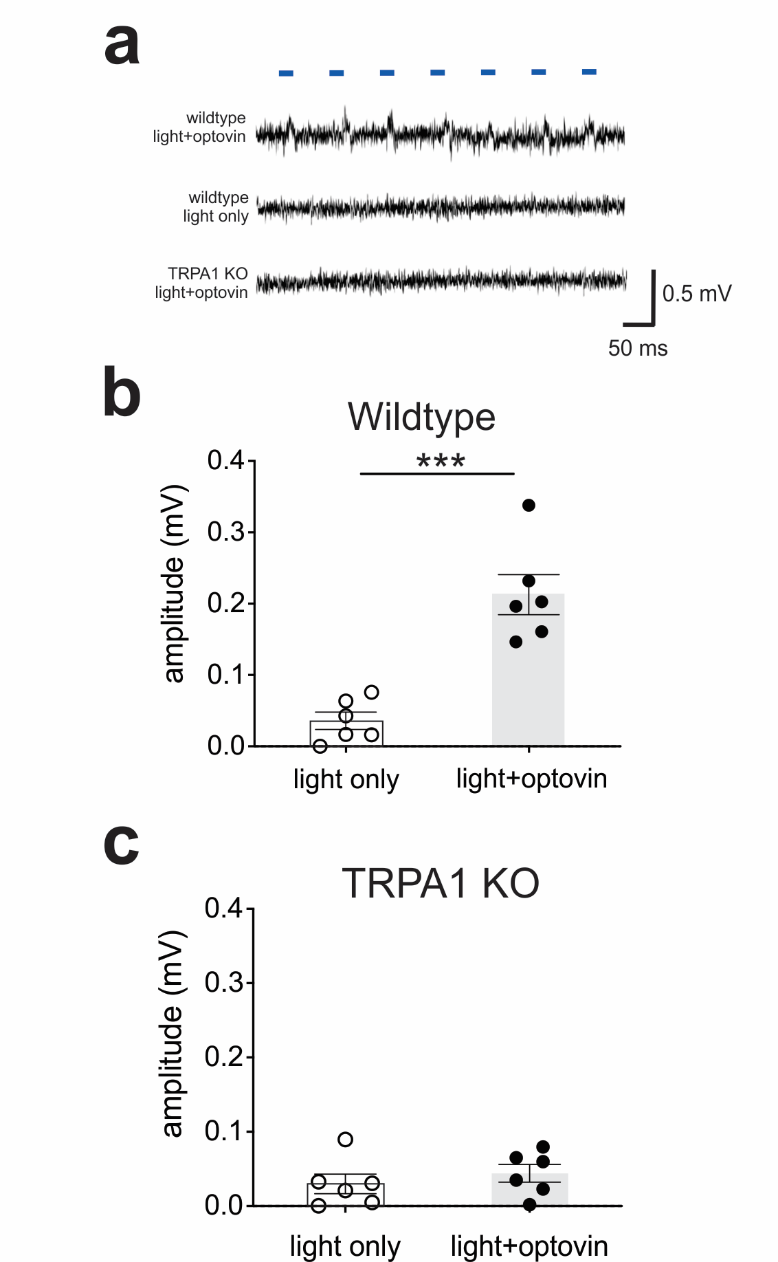
**

**Figure S6.** **Opto-pharmacological activation of TRPA1 on the vagus nerve. (a-c)** Optovin was directly administered to the exposed cervical vagus in wildtype or TRPA1 KO mice nerve prior to stimulation with light. Animals were subjected to 405 nm light (1000 mA, 10 Hz, with a 10% duty cycle) stimulation or sham stimulation. **(a)** *Optovin-induced activation of TRPA1 on cervical vagus nerve fibers.* Representative neurogram recordings of wildtype mice without optovin, wildtype mice with optovin and TRPA1 KO mice with optovin. Horizontal blue bars indicate when the light pulse was on. Optovin + light stimulation induces compound action potentials in the vagus nerve in wildtype mice but not in TRPA1 KO mice. **(b-c)** *Quantification of the amplitude of light evoked potentials.* Amplitude of the optovin + light stimulation was quantitated in wildtype and TRPA1 KO mice. **(b)** Wildtype mice with light only (n=6) or light with optovin (n=6); **(c)** TRPA1 KO mice with light (n=6) or light with optovin (n=6). Data is presented as individual mouse data point with mean ± SEM. Paired t test,*** p<0.001.

**
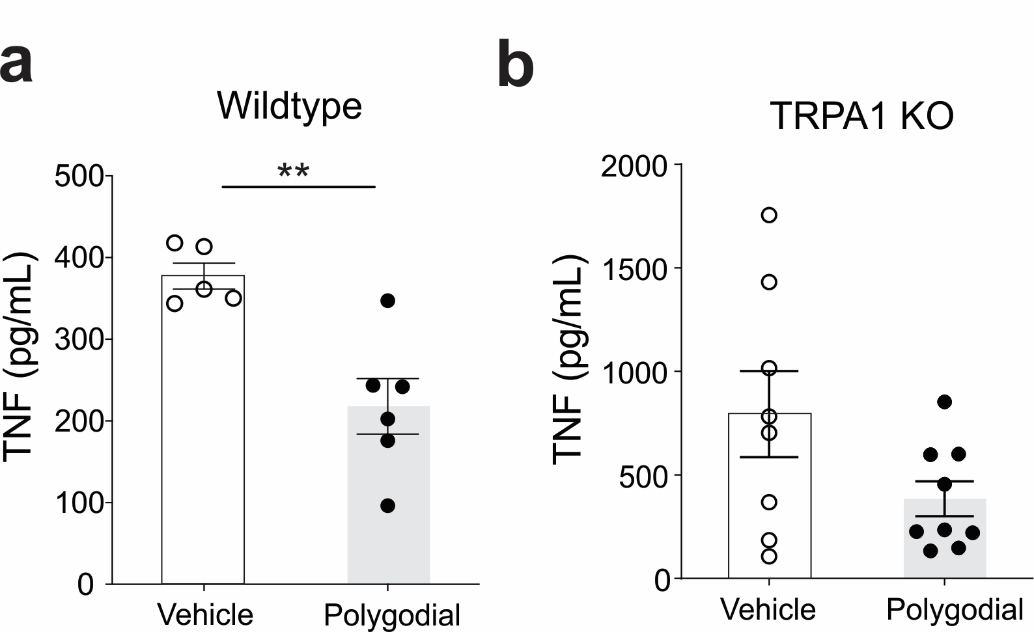
**

**Figure S7. TRPA1-specific agonist suppresses TNF production in endotoxemic wildtype mice but not in TRPA1 KO mice.** Vehicle or TRPA1 agonist, polygodial (5mg/kg, i.p.), was administered to **(a)** wildtype mice (B6.129PF, n=5-6 per group) or **(b)** TRPA1 KO mice (n=9-10 per group) 30 minutes prior to LPS administration (0.1mg/kg, i.p.). Serum was obtained 90 min after endotoxin administration, and TNF was measured by ELISA. Data is presented as individual mouse data point with mean ± SEM. Unpaired t test with Welch’s correction; ** p<0.001.


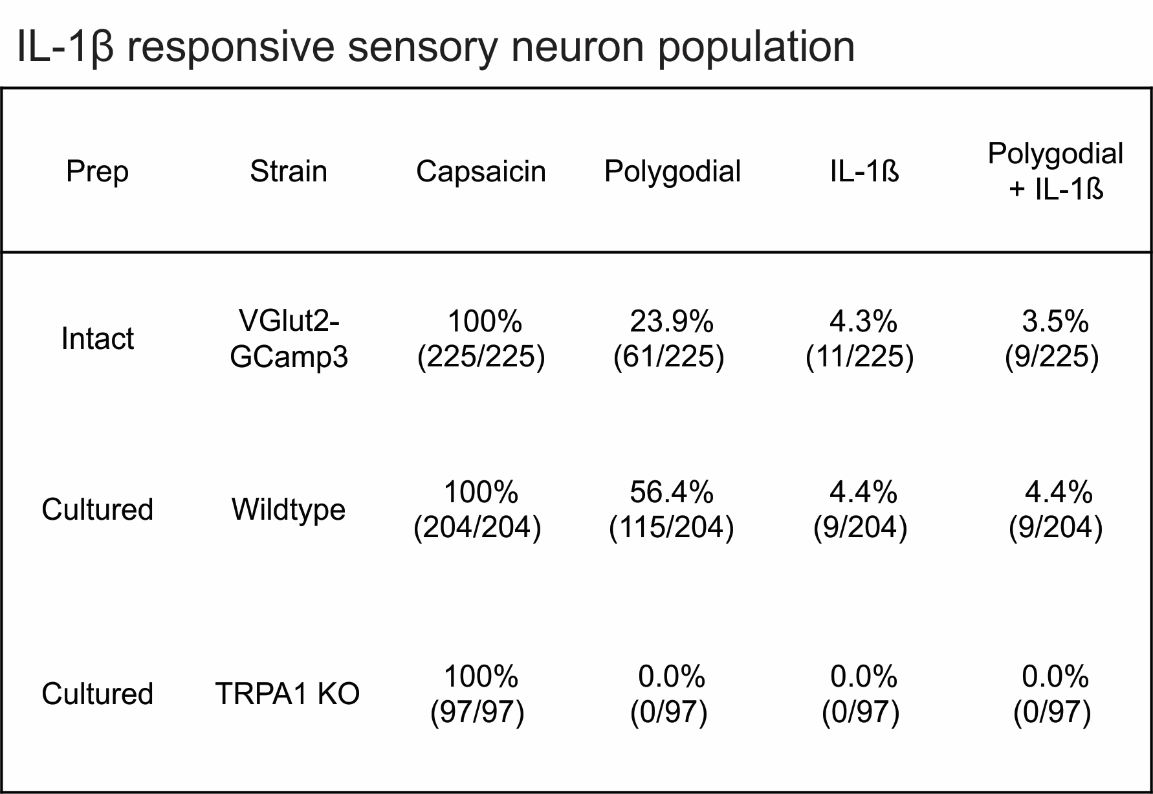


**Table S1. IL-1β responsive sensory neuron population.** Percentage of capsaicin responsive neurons that respond to polygodial, IL-1β, and both IL-1β and polygodial in intact and cultured nodose ganglion neurons isolated from Vglut2-Cre/GCaMP3, wildtype (B6.129PF), and TRPA1 KO mice.
